# Development and validation of a simple-to-use nomogram to predict liver metastasis in patients with pancreatic neuroendocrine neoplasms: a large cohort study

**DOI:** 10.1186/s12876-021-01685-w

**Published:** 2021-03-04

**Authors:** Maoen Pan, Yuanyuan Yang, Tianhong Teng, Fengchun Lu, Yanchan Chen, Heguang Huang

**Affiliations:** Department of General Surgery, Fujian Medical University Union Hospital, No.29, Xinquan Road, Fuzhou, 350001 China

**Keywords:** Pancreas, Nomogram, Neuroendocrine neoplasms, Liver metastasis, SEER databases

## Abstract

**Background:**

Liver metastasis is an important prognostic factor for pancreatic neuroendocrine neoplasms (pNENs), but the relationship between the clinical features of patients with pNEN and liver metastasis remains undetermined. The aim of this study was to establish and validate an easy-to-use nomogram to predict liver-metastasis in patients with pNEN.

**Methods:**

We obtained the clinicopathologic data of 2960 patients with pancreatic neuroendocrine neoplasms from the Surveillance, Epidemiology and End Results (SEER) database between 2010 and 2016. Univariate and multivariate logistic regression were done to screen out independent influencing factors to establish the nomogram. The calibration plots and the area under the receiver operating characteristic curve (AUC) were used to evaluate the performance of nomogram. Decision curve analysis (DCA) was applied to compare the novel model with the conventional predictive methods.

**Results:**

A total of 2960 patients with pancreatic neuroendocrine neoplasms were included in the study. Among these, 1974 patients were assigned to the training group and 986 patients to the validation group. Multivariate logistic regression identified, tumor size, grade, other site metastasis, T stage and N stage as independent risk factors. The calibration plot showed good discriminative ability in the training and validation groups, with C-indexes of 0.850 for the training cohort and 0.846 for the validation cohort. The AUC values were 0.850 (95% CI 0.830–0.869) and 0.839 (95% CI 0.812–0.866), respectively. The nomogram total points (NTP) had the potential to stratify patients into low risk, medium risk and high risk (P < 0.001). Finally, comparing the nomogram with traditional prediction methods, the DCA curve showed that the nomogram had better net benefit.

**Conclusions:**

Our nomogram has a good ability to predict liver metastasis of pancreatic neuroendocrine neoplasms, and it can guide clinicians to provide suitable prevention and treatment measures for patients with medium- and high-risk liver metastasis.

**Supplementary Information:**

The online version contains supplementary material available at 10.1186/s12876-021-01685-w.

## Background

Pancreatic neuroendocrine neoplasms (pNENs) are relatively rare, with an estimated annual incidence of approximately 3.65/10,000 people per year [1, 2]. The natural disease progression of pancreatic neuroendocrine tumors can lead to local lymph node, liver, lung, and bone metastases. Among these, liver metastases are the most common. It is reported that more than 60% of patients with pNEN have liver metastases [3]. Studies have found that liver metastasis is an important risk factor for prognosis [4]. The treatment strategy and prognosis of pNEN largely depend on whether there is liver metastasis. Therefore, early diagnosis and treatment of pNEN patients with liver metastases can significantly improve the quality of life and prognosis. Due to the lack of typical clinical manifestations of nonfunctional pNEN in the early stage, 20% to 30% of pNEN patients have liver metastases when diagnosed, which seriously affects their quality of life and long-term survival [5, 6]. Therefore, it is critical that clinicians accurately identify the risk of liver metastases in patients with pNEN for optimal treatment strategies.

The routine examination for excluding liver metastasis is a computed tomography (CT), but it has low sensitivity and specificity for microscopic liver metastasis [7]. Previous studies have shown that liver metastases from neuroendocrine tumors are correlated with a variety of clinicopathological factors, including histological type, primary site, tumor size, lymphatic invasion, and proliferative activity [8, 9]. However, the above studies are limited to some fragmentary risk factors and small sample sizes. It is essential to explore the relationship between clinicopathological factors and liver metastasis based on a large sample database and to develop a prediction model of the risk of liver metastasis in pNEN patients.

In this study, we constructed and validated a simple-to-use nomogram model. With this prediction model, clinicians can accurately identify patients with pNEN at medium and high-risk of liver metastasis patients with pNEN and provide patients with personalized prevention and treatment strategies.

## Methods

### Study population and data sources

The data were extracted from the Surveillance, Epidemiology, and End Results (SEER) database using SEER*Stat software Version 8.3.6. Data from patients with pNEN diagnosed in 2010–2016 who had complete information including age, sex, race, primary site, grade, marital status, T stage, N stage, tumor size, histology, and metastasis site, were included in the study. Pancreatic neuroendocrine neoplasms were selected on the basis of International Classification of Disease codes (ICD-O-3), including carcinoma (8150), malignant beta-cell tumor (8151), malignant alpha-cell tumor (8152), G-cell tumor (8153), VIPoma (8155), malignant somatostatinoma (8156), carcinoid tumor (8240), carcinoid tumor (8240) and atypical carcinoid tumor (8249). The exclusion criteria were as follows: (1) patients without definitive liver metastasis data; (2) patients with more than one primary cancer; and (3) patients without definitive grade and metastasis site information.

### Construction and validation of the nomogram

We randomly assigned two-thirds of our patients to the training group and the rest of them were assigned to the validation group. The chi-square tests was used to compare the baseline characteristics of the two groups. In the training group, liver metastasis risk factors were determined through the univariate logistic regression. Variates with P values less than 0.05 were used in the multivariate logistic regression analysis. Based on the coefficients of the independent risk factors in the multivariate analysis, the prediction model was visualized in the form of the nomogram. To draw this nomogram, we needed to assign a score of 0–100 to each factor. The coefficients of the above multiple logistic regression results were transformed and are shown in the form of graphs. The nomogram's ruler for each indicator was based on the index with the most influence. The greater the influence of the risk factors, the higher the nomogram score [10]. The whole process was done in R 3.6.2 software. The details of building the nomogram and R codes are provided in Additional file 1: Supplement Method 1.

The concordance index (C-index), the receiver operating characteristic curve (ROC), and the area under the curve (AUC) were used to evaluate the predictive accuracy and discrimination of the nomogram. The decision curve (DCA) [11] was used to evaluate the clinical utility of the nomogram, and compare nomogram with conventional predictive risk factors including grade, T stage, and tumor size. The details of DCA curve building and R codes were provided in Additional file 1: Supplement Method 2.

### Risk group stratification and statistical analysis

According to the characteristics of each patient's risk factors, a straight line was drawn to the "point" at the top of the model to obtain each factor score. The total score was obtained by summing the scores for all the factors. To further discriminate the risk groups of liver metastasis, the patients were categorized into low-, medium- and high-risk groups based on the nomogram total points (NTP) of every pNEN patients. The optimal two cut-off values for NTP were calculated by X-tile software. The cut-off value was then validated in the validation group. The chi-square test was used to compare all risk groups.

Statistical analysis was performed using SPSS software version 23 and R version 3.6.2 software. For all analyses, P values less than 0.05 were considered statistically significant.

## Result

### Baseline characteristics of the patients

There were 2960 eligible patients with pNEN who were included in this study. A total of 1974 patients were allocated to the training group and 986 cases were allocated to the validation group. The two groups had no significant difference in baseline characteristics (all P > 0.05) (Table 1). In the entire study group, the median age was 58 years. The majority of the patients were white (n = 2268, 76.6%) and married (n = 1814, 61.3%). The pancreatic tail was the most common site of pNEN tumors (n = 1058, 35.7%). The main pathological grade of neoplasms was G1 (n = 2068, 69.9%), followed by G2 (n = 577, 19.5%). During the whole follow-up, most of the patients were alive (81.9%) and only 535 (18.1%) patients died. There were 419 (21.2%) and 222 (22.5%) pNEN patients with liver metastases in the training group and validation group, respectively. Liver metastasis was found to be correlated with sex, primary site, grade, T stage, N stage, tumor size and other site metastasis in pNEN patients (Table 2).

**Table 1:** Baseline characteristics of the pNEN patients

n (%)	Total	Training group	Validation group	P value
	(n = 2960)	(n = 1974)	(n = 986)	
*Age*				0.105
< 65	1909 (64.5)	1293 (65.5)	616 (62.5)	
≥ 65	1051 (35.5)	681 (34.5)	370 (37.5)	
*Sex*				0.056
Male	1626 (54.9)	1060 (53.7)	566 (57.4)	
Female	1334 (45.1)	914 (46.3)	420 (42.6)	
*Race*				0.282
White	2268 (76.6)	1511 (76.5)	757 (76.8)	
Black	347 (11.7)	242 (12.3)	105 (10.6)	
Other	345 (11.7)	221 (11.2)	124 (12.6)	
*Primary site*				0.063
Head	867 (29.3)	614 (31.1)	263 (26.7)	
Body	466 (15.7)	302 (15.3)	164 (16.6)	
Tail	1058 (35.7)	695 (35.2)	353 (35.8)	
Other	569 (19.2)	363 (18.4)	206 (20.9)	
*Grade*				0.540
G1	2068 (69.9)	1386 (70.2)	682 (69.2)	
G2	577 (19.5)	374 (18.9)	203 (20.6)	
G3	315 (10.6)	214 (10.8)	101 (10.2)	
*Marital status*				0.643
Married	1814 (61.3)	1209 (61.2)	605 (61.4)	
Unmarried	513 (17.3)	335 (17.0)	178 (18.1)	
Other	633 (21.4)	430 (21.8)	203 (20.6)	
*T stage*				0.889
T1	893 (30.2)	591 (29.9)	302 (30.6)	
T2	948 (32.0)	635 (32.2)	313 (31.7)	
T3	780 (26.4)	526 (26.6)	254 (25.8)	
T4	147 (5.0)	93 (4.7)	54 (5.5)	
Unspecific	192 (6.5)	129 (6.5)	63 (6.4)	
*N stage*				0.245
N0	2069 (69.9)	1364 (69.1)	705 (71.5)	
N1	773 (26.1)	534 (27.1)	239 (24.2)	
Unspecific	118 (4.0)	76 (3.9)	42 (4.3)	
*Tumor size*				0.825
< 2	880 (29.7)	592 (30.0)	288 (29.2)	
2–4	958 (32.4)	636 (32.2)	322 (32.7)	
≥ 4	965 (32.6)	637 (32.3)	328 (33.3)	
Unspecific	157 (5.3)	109 (5.5)	48 (4.9)	
*Other site metastasis*				0.949
Yes	109 (3.7)	73 (3.7)	36 (3.7)	
No	2851 (96.3)	1901 (96.3)	950 (96.3)	
*Functional status*				0.800
Functional	223 (7.5)	147 (7.4)	76 (7.7)	
Nonfunctional	2737 (92.5)	1827 (92.6)	910 (92.3)	
*Liver metastasis*				0.422
Yes	641 (21.7)	419 (21.2)	222 (22.5)	
No	2319 (78.3)	1555 (78.8)	764 (77.5)

**Table 2:** The relationship of pNEN patients with liver metastases and clinicopathological factors in the training group and the validation group

Characteristic	Training group			P	Validation group			P
	Total (%)	Live-metastasis			Total (%)	Live-metastasis		
		Yes (n = 419)	NO (n = 1555)			Yes (n = 222)	No (n = 764)	
*Age*				0.867				0.055
< 65	1293 (65.5)	273 (65.2)	1020 (65.6)		616 (62.5)	126 (56.8)	490 (64.1)	
≥ 65	681 (34.5)	146 (34.8)	535 (34.4)		370 (37.5)	96 (43.2)	274 (35.9)	
*Sex*				0.036				0.025
Male	1060 (53.7)	244 (58.2)	816 (52.5)		566 (57.4)	142 (64.0)	424 (55.5)	
Female	914 (46.3)	175 (41.8)	739 (47.5)		420 (42.6)	80 (36.0)	340 (44.5)	
*Race*				0.077				0.064
White	1511 (76.5)	333 (79.5)	1178 (75.8)		757 (76.8)	183 (82.4)	574 (75.1)	
Black	242 (12.3)	52 (12.4)	190 (12.2)		105 (10.6)	16 (7.2)	89 (11.6)	
Other	221 (11.2)	34 (8.1)	187 (12.0)		124 (12.6)	23 (10.4)	101 (13.2)	
*Primary site*				< 0.001				0.041
Head	614 (31.1)	132 (31.5)	482 (31.0)		263 (26.7)	61 (27.5)	202 (26.4)	
Body	302 (15.3)	29 (6.9)	273 (17.6)		164 (16.6)	27 (12.2)	137 (17.9)	
Tail	695 (35.2)	151 (36.0)	544 (35.0)		353 (35.8)	75 (33.8)	278 (36.4)	
Other	363 (18.4)	107 (25.5)	256 (16.5)		206 (20.9)	59 (26.6)	147 (19.2)	
*Grade*				< 0.001				< 0.001
G1	1386 (70.2)	188 (44.9)	1198 (77.0)		682 (69.2)	99 (44.6)	583 (76.3)	
G2	374 (18.9)	102 (24.3)	272 (17.5)		203 (20.6)	63 (28.4)	140 (18.3)	
G3	214 (10.8)	129 (30.8)	85 (5.5)		101 (10.2)	60 (27.0)	41 (5.4)	
*Marital status*				0.351				0.670
Married	1209 (61.2)	244 (58.2)	965 (62.1)		605 (61.4)	131 (59.0)	474 (62.0)	
Unmarried	335 (17.0)	78 (18.6)	257 (16.5)		178 (18.1)	44 (19.8)	134 (17.5)	
Other	430 (21.8)	97 (23.2)	333 (21.4)		203 (20.6)	47 (21.2)	156 (20.4)	
*T stage*				< 0.001				< 0.001
T1	591 (29.9)	15 (3.6)	576 (37.0)		302 (30.6)	7 (3.2)	295 (38.6)	
T2	635 (32.2)	120 (28.6)	515 (33.1)		313 (31.7)	62 (27.9)	251 (32.9)	
T3	526 (26.6)	152 (36.3)	374 (24.1)		254 (25.8)	82 (36.9)	172 (22.5)	
T4	93 (4.7)	52 (12.4)	41 (2.6)		54 (5.5)	32 (14.4)	22 (2.9)	
Unspecific	129 (6.5)	80 (19.1)	49 (3.2)		63 (6.4)	39 (17.6)	24 (3.1)	
*N stage*				< 0.001				< 0.001
N0	1364 (69.1)	182 (43.4)	1182 (76.0)		705 (71.5)	112 (50.5)	593 (77.6)	
N1	534 (27.1)	198 (47.3)	336 (21.6)		239 (24.2)	82 (36.9)	157 (20.5)	
Unspecific	76 (3.9)	39 (9.3)	37 (2.4)		42 (4.3)	28 (12.6)	14 (1.8)	
*Tumor size*				< 0.001				< 0.001
< 2	592 (30.0)	18 (4.3)	574 (36.9)		288 (29.2)	6 (2.7)	282 (36.9)	
2—4	636 (32.2)	102 (24.3)	534 (34.3)		322 (32.7)	67 (30.2)	255 (33.4)	
≥ 4	637 (32.3)	238 (56.8)	399 (25.7)		328 (33.3)	120 (54.1)	208 (27.2)	
Unspecific	109 (5.5)	61 (14.6)	48 (3.1)		48 (4.9)	29 (13.1)	19 (2.5)	
*Other site metastasis*				< 0.001				< 0.001
Yes	73 (3.7)	52 (12.4)	21 (1.4)		36 (3.7)	24 (10.8)	12 (1.6)	
No	1901 (96.3)	367 (87.6)	1534 (98.6)		950 (96.3)	198 (89.2)	752 (98.4)	
*Functional status*				0.426				0.068
Functional	147 (7.4)	35 (8.4)	112 (7.2)		76 (7.7)	24 (10.8)	52 (6.8)	
Nonfunctional	1827 (92.6)	384 (91.6)	1443 (92.8)		910 (92.3)	198 (89.2)	712 (93.2)	

### Independent risk factors and nomogram construction

**Fig. 1:**
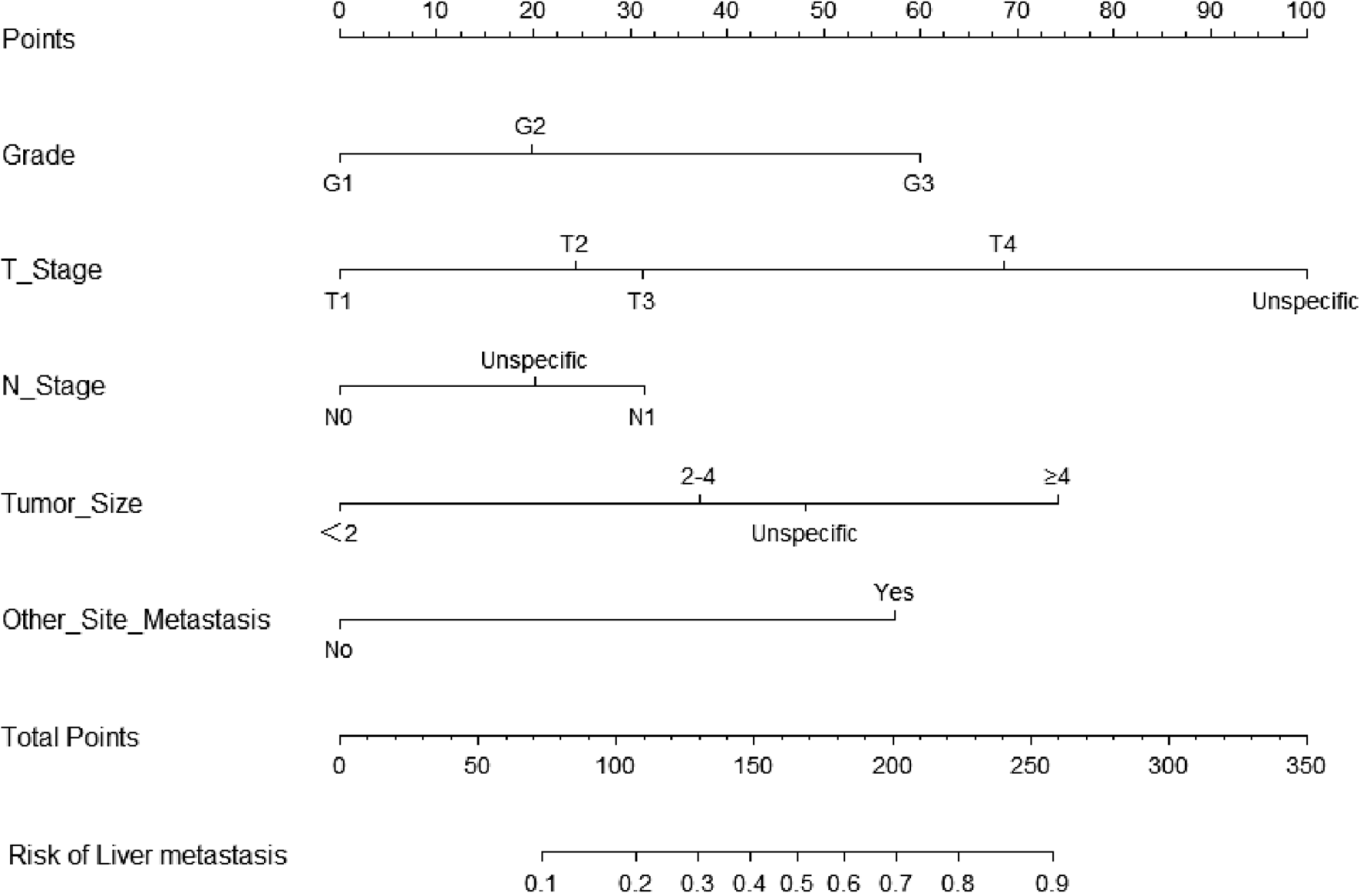
Nomogram for predicting the risk of liver metastasis in patients with pancreatic neuroendocrine neoplasms. Assign points to each risk factor by drawing a line up from the corresponding value to the point line. The total points of all risk factors are summed and are found on the total points line. A line is drawn down to read the corresponding prediction of liver metastasis risk in pNEN patients

Univariate regression analysis was used to screen the risk factors for liver metastasis. The significant risk variables were included in the multivariate regression analysis. The results of multivariate logistic regression analysis showed that grade, T stage, N stage, tumor size, and other site metastasis were independent risk factors for liver metastasis (Table 3). All the above variables were used to establish the nomogram model (Fig. 1). In this model, it was found that grade, T stage and tumor size had the greatest impact on liver metastasis, followed by N stage and other site metastasis. The probability of liver metastasis in each pNEN patient can be computed by adding up the corresponding scores of all the independent risk factors.

**Table 3:** Univariate and multivariate logistic analyses of liver metastasis in pNEN patients

Characteristic		Univeriate analysis			Multivariate analysis		
		OR	95%CI	P value	OR	95%CI	P value
Age	≤ 65/ > 65	1.020	0.813–1.279	0.867			
Sex	Male/female	0.792	0.637–0.985	0.036	0.954	0.737–1.236	0.723
Race	White/black/other	0.835	0.703–0.991	0.039	0.923	0.756–1.126	0.429
Tumor site	Head/body/tail/other	1.165	1.056–1.285	0.002	1.104	0.987–1.235	0.083
Grade	G1/G2/G3	2.975	2.562–3.455	< 0.001	2.073	1.752–2.451	< 0.001
Marital status	Married/unmarried/other	1.085	0.953–1.234	0.219			
T stage	T1/T2/T3/T4/unspecific	2.437	2.188–2.714	< 0.001	1.461	1.256–1.699	< 0.001
N stage	N0/N1/unspecific	3.180	2.641–3.830	< 0.001	1.604	1.279–2.010	< 0.001
Tumor size	< 2/2–4/ ≥ 4/unspecific	3.335	2.869–3.878	< 0.001	1.927	1.577–2.354	< 0.001
Other site metastasis	Yes/no	0.097	0.057–0.162	< 0.001	0.241	0.133–0.435	< 0.001
Functional status	Functional/nonfunctional	0.852	0.573–1.265	0.426

### Nomogram validation and risk classification

**Fig. 2:**
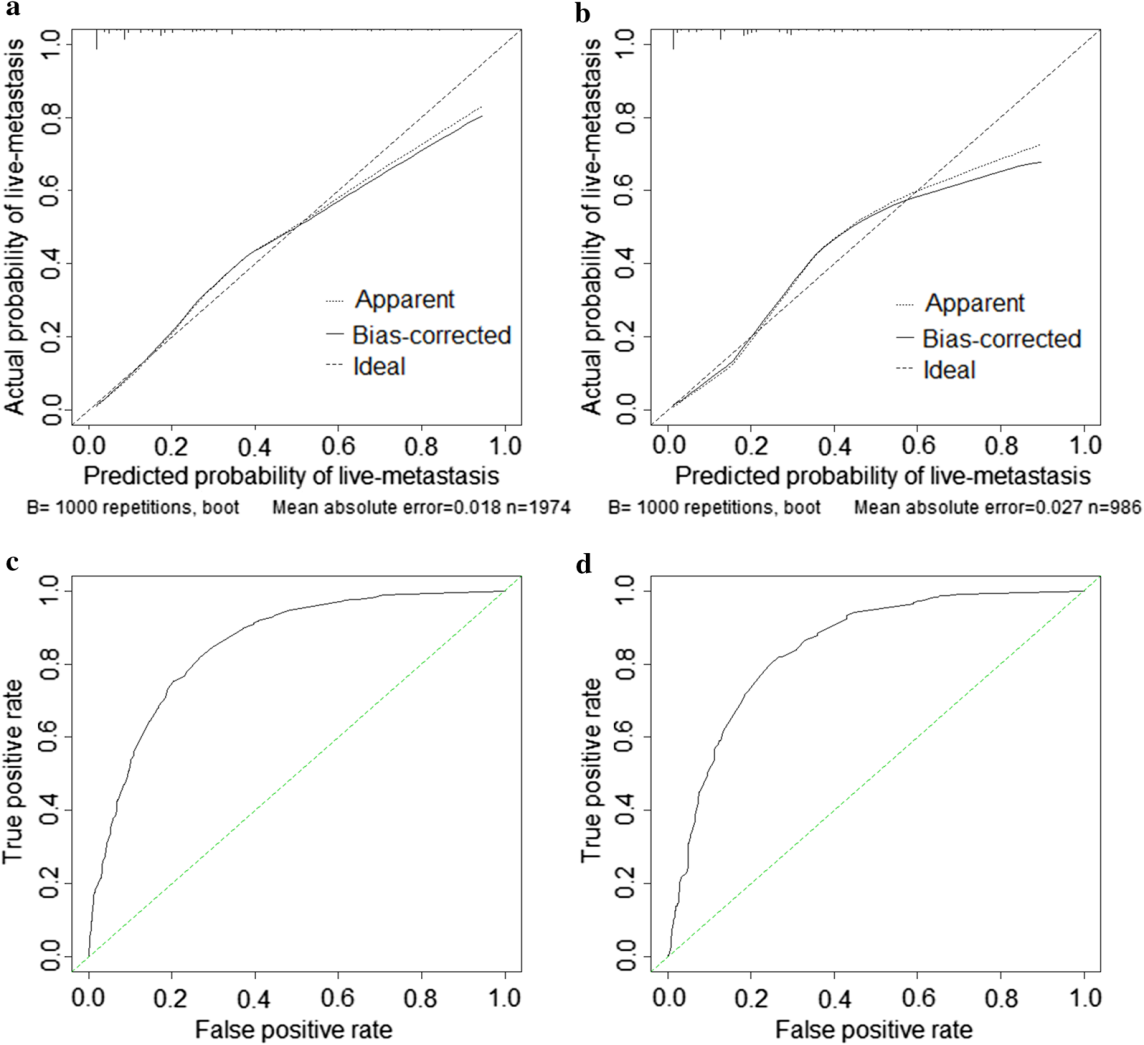
The calibration plots and ROC curves in the training cohort (**A** and **C**, respectively) and the validation cohort (**B** and **D**, respectively)

**Fig. 3:**
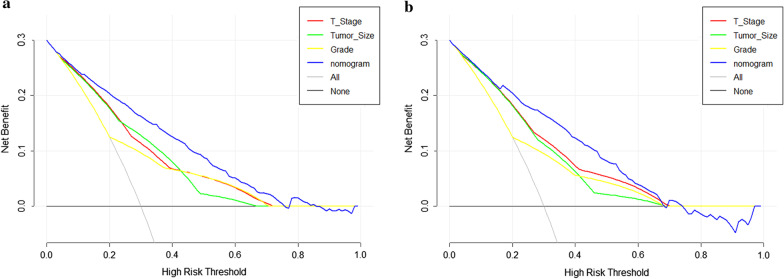
DCA for the nomogram and the conventional forecasting methods including grade, T-stage, and tumor size in the training (**A**) and validation groups (**B**). The x-axis shows the threshold probabilities. The y-axis measures the net benefit, which is calculated by adding the true positives and subtracting the false positives. The horizontal solid black line: assumes no liver metastasis will happen; the solid grey line: assumes all patients will experience tumor liver metastasis. In DCA, the nomogram yielded a superior clinical net benefit compared with the conventional forecasting methods across a range of threshold probabilities

The calibration plot showed good agreement in the training and validation group (Fig. 2A, B). The C-index of liver metastasis prediction was 0.850 and 0.846 in the training and validation group, respectively. When the ROC curves were plotted, the training group had an AUC of 0.850 (95% CI 0.830–0.869), which was verified in the validation group (AUC = 0.839, 95% CI 0.812–0.866) (Fig. 2C, D). Decision curve analysis (DCA) was done next (Fig. 3), which is a novel method that can evaluate the clinical practicality of models. The results showed that the nomogram had satisfactory net benefits among most of the threshold probabilities in both groups. Compared with conventional predictive methods, our nomogram was more exact in predicting liver metastasis.

**Fig. 4:**
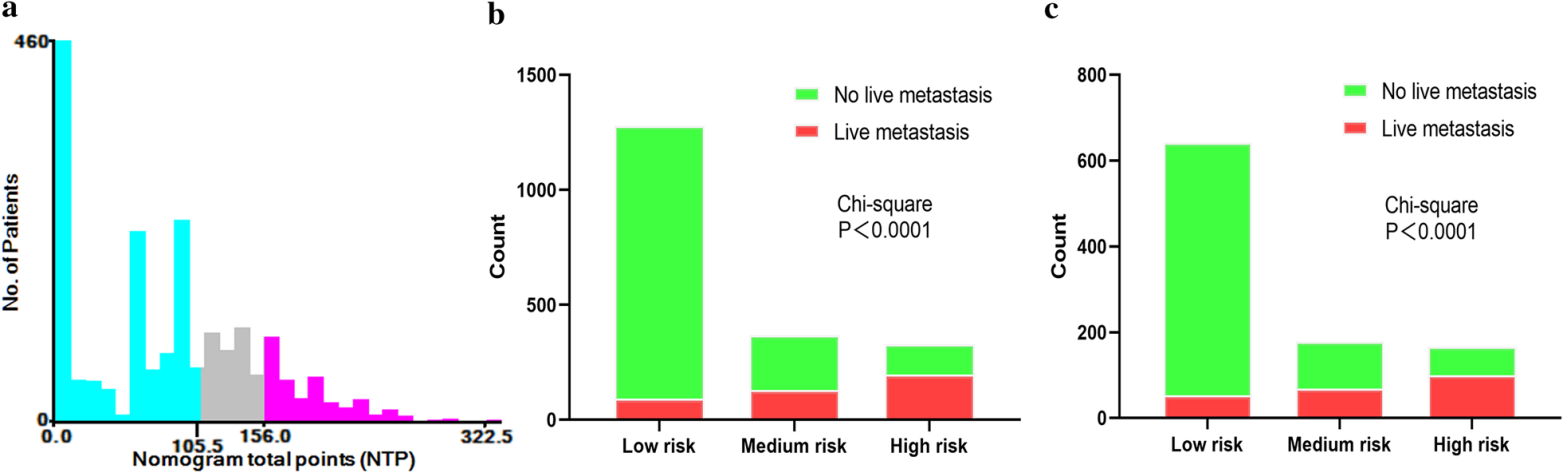
X-tile software was used to find the best cut-off value (**A**). The risk-classification performance of the nomogram in the training (**B**) and validation groups (**C**)

The training group was divided into three subgroups based on the two optimal NTP cut-off values. According to the X-tile calculation results, the optimal cut-off values were 105.5 and 156.0 respectively (Fig. 4A). The patients were divided into low-risk (NTP < 105.5, n = 1278 (64.7%)), medium-risk (105.5 ≤ NTP < 156.0, n = 368 (18.6%)) and high-risk subgroups (NTP ≥ 156.0, n = 328 (16.6%)). The same cut-off values were used for grouping in the validation group. Notably, the high-risk pNEN patients were more likely to have liver metastases in both groups (P < 0.05) (Fig. 4B, C).

## Discussion

Although the natural history of many pancreatic neuroendocrine tumors is characterized by slow progression and inertia, there are still patients with metastasis during the course of the disease, especially liver metastasis. For patients with resectable pNEN with liver metastases, active surgical resection of primary and liver metastases should be the preferred treatment. Previous studies have reported that surgical resection of primary and metastatic lesions could improve quality of life and prolong survival, with a 5-year survival rate of 60–80% [12–16]. However, due to the limited sensitivity of the current imaging modalities, early pNEN patients with liver metastasis have a high rate of missed diagnosis, which makes the patients lose their best chance of radical surgical resection when they are diagnosed. Liver biopsy has a high diagnosis rate, but it increases the risk of distant metastasis and leads to reduced survival time [17]. Therefore, a noninvasive and simple-to-use method is required for predicting the likelihood of liver metastasis in patients with pNEN. In our study, a novel nomogram was developed for predicting the probability of liver metastasis of pNEN based on a large database. The results demonstrated that the nomogram model is significantly discriminative and thus provides an individualized prediction of the probability of liver metastasis.

Our study mainly focussed on the clinical characteristics of pNEN patients with liver metastasis, and demonstrated that grade, T stage, N stage, tumor size, and other site metastasis were independent risk factors for liver metastasis. The G1-2 group had a higher percentage of pNEN patients with liver metastases (70.5%) than the other groups. This result is similar to that of Ruzzenente (81.9%) [18]. In addition, Spolverato [19] found that nonfunctional and moderate-to-poor tumors were more likely to have liver metastases. We speculate the reason that the G1-2 non-functional tumor easily neglected in the early stage due to the lack of obvious clinical symptoms, and the tumor is already in advanced stage when diagnosed. Previous studies have shown that the main cause of liver metastases is vascular invasion [20]. During hematogenous metastasis, the liver is the first filter for tumor cell invasion. In this study, we found that the size and T stage of the primary tumor were closely related to the infiltration of neuroendocrine tumor cells into the liver. The size of the tumor is directly related to the T stage. The larger the primary tumor size, the more aggressive it is towards surrounding organs or blood vessels. This study also confirmed that the larger the tumor and the higher the T stage, the greater the probability of liver metastasis.

Apart from the route of hematogenous metastasis, pancreatic neuroendocrine tumor may also metastasize to distant sites via lymphatic pathways. In our study, LN metastasis was identified as an independent risk factor in predicting liver metastasis. Positive lymph nodes are a common sign before distant metastasis, which has been demonstrated in other tumors [21, 22]. In our study, 47.3% of patients with liver metastases had positive lymph nodes. Therefore, more attention should be paid to the presence of metastasis in the liver and other sites in patients with positive lymph nodes. Besides liver metastasis, there were also other distant site metastases (bone, lung, brain). In this study, more than 72.2% of pNEN patients with other site metastases also had liver metastases. This result reveals that there are probably other metastases when liver metastases are found. This finding is consistent with other studies [23–25].

The advice given to the patient and the choice made among treatment options are based on the assessment of the individual's prognosis and risk [26]. Nomograms are graphical representations of statistical prediction models that predict the probability of an event occurring [27]. Thus, the variables contained in the nomogram should be easy to obtain and measure. In this study, we developed a nomogram to predict live metastasis in patients with pNEN. Our nomogram model has been shown to have good discernment with high C-indexes and AUCs, in both groups. Finally, DCA curves were generated to show that the nomogram could be used to obtain a better net benefit within the derived probabilities than traditional prediction methods [26].

There are some limitations to this study. The major limitation of our study is the lack of important variables, such as surgical margin, Ki-67 and other molecular biomarkers. The Ki-67 index and surgical margin play an important role in the prognosis of pNEN [28]. Unfortunately, the absence of Ki-67 and surgical margins in the SEER database made it impossible to assess its role in predicting liver metastasis of pNEN. Second, our nomogram has been verified to have excellent prediction capabilities, but further external validation based on a large multicenter data cohort is still required. Finally, since the SEER database is a retrospective database, selection bias cannot be completely avoided. Therefore, bootstrapping with 1000 resamples was performed in this study to minimize bias.

## Conclusion

In conclusion, we successfully created and validated a simple-to-use nomogram for predicting the probability of liver metastasis in pNEN patients. This model has good predictive power and it is easy for the clinician to use. By assessing the risk of liver metastasis, clinicians could realize individualized treatment and take necessary preventive measures to reduce the risks borne by patients and improve their quality of life and prognosis.

## Supplementary Information

**Additional file 1.** The detail methods of nomogram and DCA curve construction.

## Data Availability

The datasets analyzed during the current study are available in the SEER repository (https://seer.cancer.gov/).

## Abbreviations

pNEN: Pancreatic neuroendocrine neoplasms
NTP: Nomogram total points
ROC: Receiver operating characteristic
ICD-O-3: International classification of diseases for oncology, third edition
SEER: Surveillance, epidemiology, and end results
LN: Lymph node
AUC: The area under the curve
DCA: The decision curve
