# Supplementary material for: Development and validation of a simple-to-use nomogram to predict liver metastasis in patients with pancreatic neuroendocrine neoplasms: a large cohort study
Source: BMC Gastroenterol. 2021 Mar 4;21:101. doi: 10.1186/s12876-021-01685-w (PMC7934499; doi:10.1186/s12876-021-01685-w)
Supplement: Supplementary file 1 — Additional file 1. The detail methods of nomogram and DCA curve construction. [file 12876_2021_1685_MOESM1_ESM.docx]

**Supplement information for**

**Development and validation of a simple-to-use nomogram to predict liver metastasis in patients with pancreatic neuroendocrine neoplasms: a large cohort study**

Maoen Pan^1,#^, Yuanyuan Yang^1,#^, Tianhong Teng^1^, Fengchun Lu^1^, Yanchan Chen^1^, Heguang Huang^1,*^.

^1^ Department of General Surgery, Fujian Medical University Union Hospital, Fuzhou 350001, China.

*Corresponding author :

Heguang Huang

Department of General Surgery, Fujian Medical University Union Hospital, No.29, Xinquan Road, Fuzhou 350001, China.
E-mail address: [heguanghuang123@163.com](mailto:heguanghuang123@163.com).

^#^ These authors contributed equally to this work.

Supplement Methods 1, 2:

S- Method 1

**The detail methods of nomogram construction.**

The establishment of the model needs to be completed by R 3.6.2 and RStudio software. The following is the codes of R language to establish the model:

**# Installation package**

install.packages("rms")

install.packages("Hmisc")

install.packages("lattice")

install.packages("survival")

install.packages("Formula")

install.packages("ggplot2")

install.packages("foreign")

install.packages("SparseM")

install.packages("pROC")

install.packages('rmda')

**# Loader package**

library(foreign)

**## Read “rms” packages and other packages**

library(Hmisc); library(grid); library(lattice); library(Formula); library(ggplot2); library(rms); library(SparseM);

**#** [**Reading**](javascript:;) [**data**](javascript:;)

bc<-read.spss("D:/shuju/pnen(training).sav",use.value.labels=F, to.data.frame=T)

**# Delete missing values**

bc <- na.omit(bc)

**# Load the “rms” package**

library(rms)

**#** [**Data**](javascript:;) [**package**](javascript:;)

bc$Grade<- factor(bc$Grade, levels = c(1,2,3),labels = c("G1","G2","G3"))

bc$T_Stage<-factor(bc$T,levels=c(1,2,3,4,5),labels=c("T1","T2","T3","T4","Unspecific"))

bc$N_Stage<- factor(bc$N,levels = c(1,2,3),labels = c("N0","N1","Unspecific"))

bc$Tumor_Size<- factor(bc$Size,levels = c(1,2,3,4),labels = c("＜2","2-4","≥4","Unspecific"))

bc$Other_Site_Metastasis<-factor(bc$othersite, levels =c(1,2),labels = c("Yes","No"))

dd <- datadist(bc)

options(datadist="dd")

**# Generated function**

f<-lrm(liver_metastasis~Grade+T_Stage+N_Stage+Tumor_Size+Other_Site_Metastasis, data =bc)

**#** [**Build**](javascript:;) **nomogram**

nom <- nomogram(f, fun= function(x)1/(1+exp(-x)), # or fun=plogis

lp=F, funlabel=" Risk of Liver metastasis")

**#Plot nomogram**

plot(nom)

**S-Method 2**

The detail methods of DCA curve.

The probability of patient i being diagnosed with liver metastasis was denoted as Pi; When Pi reaches a certain threshold (denoted as Pt ), it is defined as positive. Applying these steps to our data gives DCA curves. **[1]**

1. Chose a value for Pt.
2. Calculate the number of true- and false-positive results using pt as the cut-point for determining a positive or negative result.
3. Calculate the net benefit of the prediction model.
4. Vary pt over an appropriate range and repeat steps 2 – 3.
5. Plot net benefit on the y axis against pt on the x axis.
6. Repeat steps 1 – 5 for each model under consideration.
7. Repeat steps 1 – 5 for the strategy of assuming all patients are positive
8. Draw a straight line parallel to the x-axis at y=0 representing the net benefit associated with the strategy of assuming that all patients are negative.

The establishment of DCA curve needs to be completed by R 3.6.2 and RStudio software. The following is the code of R language to establish DCA curve.

**library(rmda)**

T_Stage<- decision_curve(liver_metastasis ~T_Stage,data= bc,

family = binomial(link ='logit'),

thresholds= seq(0,1, by = 0.01),

confidence.intervals = 0.95,

study.design = 'case-control',

population.prevalence = 0.3)

Tumor_Size<- decision_curve(liver_metastasis ~Tumor_Size,data= bc,

family = binomial(link ='logit'),

thresholds= seq(0,1, by = 0.01),

confidence.intervals = 0.95,

study.design = 'case-control',

population.prevalence = 0.3)

Grade<- decision_curve(liver_metastasis ~Grade,data= bc,

family = binomial(link ='logit'),

thresholds= seq(0,1, by = 0.01),

confidence.intervals = 0.95,

study.design = 'case-control',

population.prevalence = 0.3)

nomogram<-decision_curve(liver_metastasis~Grade+T_Stage+N_Stage+Tumor_Size+Other_Site_Metastasis, data = bc,family = binomial(link ='logit'),

thresholds = seq(0,1, by = 0.01),

confidence.intervals= 0.95,

study.design = 'case-control',

population.prevalence= 0.3)

List<- list(T_Stage,Tumor_Size,Grade,nomogram)

**#Plot curve**

plot_decision_curve(List,curve.names=c('T_Stage','Tumor_Size','Grade','nomogram'),

cost.benefit.axis =FALSE,col=c('red','green','yellow','blue'),

confidence.intervals=FALSE,

standardize = FALSE)

**Reference**

1. Vickers AJ, Elkin EB: Decision curve analysis: a novel method for evaluating prediction models. MED DECIS MAKING 2006, 26(6):565-574.
